# Perovskite-WS_2_ Nanosheet Composite Optical Absorbers on Graphene as High-Performance Phototransistors

**DOI:** 10.3389/fchem.2019.00257

**Published:** 2019-05-08

**Authors:** Dan-Dan Zhang, Rong-Mei Yu

**Affiliations:** ^1^Jiangsu Key Laboratory for Carbon-Based Functional Materials and Devices, Institute of Functional Nano and Soft Materials (FUNSOM), Soochow University, Suzhou, China; ^2^College of Physics and Electronic Engineering, Nanyang Normal University, Nanyang, China

**Keywords:** reduced graphene oxide, perovskite, WS_2_, bulk heterojunction, photodetection

## Abstract

High-responsivity phototransistors with a structure of perovskite-WS_2_ nanosheet composite optical absorber and a reduced graphene oxide (rGO) channel layer is demonstrated via a facile and low-cost solution-processing method. The WS_2_ nanosheets are dispersed within the perovskite matrix, forming the perovskite-WS_2_ bulk heterojunction (BHJ). The hybrid phototransistor exhibits excellent figures of merit including high photoresponsivity of 678.8 A/W, high specific detectivity of 4.99 × 10^11^ Jones, high EQE value of 2.04 × 10^5^% and rapid response to photoswitching. The high photoresponsivity could be attributed to the WS_2_ nanosheets induced photo-generated electron-hole separation promotion effects due to the selective electron trapping effects in the WS_2_ nanosheets, together with the high carrier mobility of the rGO channel. This work provides a promising platform for constructing high-responsivity photodetectors.

## Introduction

Graphene has attracted extensive interests for photodetection owing to its ultrahigh carrier mobility and wavelength independent broadband light absorption (Novoselov et al., 2005; Qiao et al., 2015; Han et al., 2019). Impressive progresses have been made in graphene-based photodetectors as promising candidates to replace traditional silicon-based photodetectors. However, the ~2.3% incident light absorption of intrinsic single-layer graphene prevented efficient photocarrier generation or accumulation, resulting in low responsivity and photoconductive gain (Xia et al., 2009; Zhang et al., 2013; Yu et al., 2017). Hybridization of graphene with photosensitizer has been proved to be an effective approach to obtain high-responsivity graphene based photodetectors (Konstantatos et al., 2012; Lee et al., 2014; Xu et al., 2014; Bessonov et al., 2017; Ni et al., 2017). This graphene/photosensitizer heterostructure exhibits the superior light absorption and high charge mobility for achieving high-responsivity photodetectors. In the field-effect transistors (FETs) utilizing the graphene/photosensitizer heterostructure as channel, the photo-generated electron-hole pairs in the photosensitizer under light will be transferred to graphene. The remained carriers in the photosensitizer produces a photogating effect in graphene, and the change in the conductivity of graphene depends on the net amount of carriers in the photosensitizer. Therefore, a promising strategy for enhancing the photodetection performance is to reduce the carrier recombination within the photosensitizer and transfer one type of carriers to graphene while the other type is trapped in the photosensitizer.

Recently, organometal halide perovskite (CH_3_NH_3_PbX_3_, X = I, Br, Cl) has emerged as a promising photosensitizer due to its excellent optical and electrical properties and ease of solution-processing (Liu et al., 2013; Wehrenfenning et al., 2013; Zuo et al., 2016). Combined with the high carrier mobility of graphene, perovskite/graphene heterostructures have been widely explored in perovskite/graphene field-effect transistor (perovskite/GFET) phototransistors toward high-responsivity (Lee et al., 2014; Spina et al., 2015; Wang et al., 2015; Sun et al., 2016; Bessonov et al., 2017; Qian et al., 2017; Peng et al., 2018). Tremendous efforts have also been made to promote the electron-hole separation and selective charge trapping according to the detection mechanism (Qian et al., 2017; Qin et al., 2017; Xie, 2017; Peng et al., 2018). For example, Yan et al. reported a perovskite/ P3HT/ graphene multilayer heterostructure phototransistor due to the promoted photo-generated electron-hole separation and electron trapping in the perovskite layer (Xie, 2017). Nevertheless, the photo-generated electron-hole recombination within the perovskite layer before the interfacial effects would still limit the photoresponse. Moreover, the reported perovskite/GFET devices are mainly based on mechanically exfoliated or chemically vapor deposited (CVD) single and few layered graphene (Lee et al., 2014; Spina et al., 2015; Wang et al., 2015; Sun et al., 2016; Bessonov et al., 2017), and the fabrication processes are expensive and complicated. Therefore, it is necessary to design an efficient device structure with improved performances by a cheap and convenient technique.

In this work, we demonstrate a solution-processed phototransistor with a hybrid channel consisting of perovskite/WS_2_ nanosheet composite optical absorbers and reduced graphene oxide (rGO), which exhibits excellent photodetection behaviors. The WS_2_ nanosheets are well-dispersed within the perovskite matrix, forming the perovskite/WS_2_ nanosheet bulk heterojunction (BHJ) layer. The WS_2_ nanosheets can effectively promote the photo-generated electron-hole separation within the BHJ film owing to the selective electron trapping effects in the WS_2_ nanosheets, and more photo-induced holes are transferred to the rGO channel for improved photodetection. This hybrid phototransistor shows a fast photoresponse with a high photoresponsivity of 678.8 A/W, high specific detectivity of 4.99 × 10^11^ Jones and high EQE value of 2.04 × 10^5^%, which are about 3.5 times enhancement compared with the perovskite-only device. The results provide an alternative strategy of constructing BHJ films as the photosensitizer toward high-performance photodetectors.

## Experimental Section

Graphene oxide (GO) solution (0.5 mg/mL) and WS_2_ nanosheets were obtained from Nanjing XFNANO Materials Tech. Co., Ltd. CH_3_NH_3_I, PbI_2_, 3-aminopropyltriethoxysilane, dimethyl sulfoxide and N, N-dimethylformanide were purchased from Alfa Aesar. Toluene was purchased from Aladdin. All these materials and chemicals were used without further purification.

(100) oriented N-type heavily doped Si wafers with 90 nm SiO_2_ layers were sequentially cleaned in acetone, anhydrous ethanol and isopropyl alcohol (IPA) for 15 min via ultrasonic treatment, followed by a 20-min UV-Ozone treatment. The cleaned SiO_2_/Si substrates were soaked in a 1 vol.% APTES toluene solution for 30 min in order to assemble the APTES monolayer on the surface. Graphene oxide (GO) solution was spin-coated onto the SiO_2_/Si substrates via the APTES monolayer and rinsed by DI water, followed by mild reduction into reduced GO (rGO) with a 30-min 150°C annealing treatment in air ambient. The CH_3_NH_3_PbI_3_ perovskite precursor was prepared by dissolving CH_3_NH_3_I:PbI_2_ (1 mmol:1 mmol) in 1 mL coalescing solvents of DMSO: DMF 3:7 (*v/v*) and stirring at 60 °C overnight. For the perovskite-WS_2_ nanosheets composite precursor, the WS_2_ nanosheets were mixed with the above CH_3_NH_3_PbI_3_ solution with a concentration of 0.05 wt.%. Subsequently, for fabricating pristine CH_3_NH_3_PbI_3_ and CH_3_NH_3_PbI_3_-WS_2_ composite photosensitizer films, the perovskite precursor solution was spin coated onto the rGO channel layer at 4,000 rpm for 40 s, during which chlorobenzene was dropped serving as the anti-solvent. The as-prepared films were annealed at 100°C for 10 min. Finally, 70 nm Au source/drain (S/D) electrodes were defined with a shadow mask and deposited by thermal evaporation. The effective detection area of the phototransistor is 30 × 500 μm^2^.

The Raman characterization of rGO films was carried out with a Raman spectrometer (WITec, 300R). The XRD patterns were measured with a PANalytical 80 equipment with Cu *K*α radiation. The UV-vis spectra were measured by using the photometer (Perkin Elmer, Lambda 750). The steady-state photoluminescence (PL) spectra were recorded on a spectrofluorometer (HORIBA Scientific, FluoroMax-4) with a 532 nm laser. The photodetection performances of the pristine and hybrid phototransistor were studied using an Agilent B1500A semiconductor analyzer in air at room temperature. The monochromic light with different wavelengths was induced by a light-emitting diode illuminant.

## Results and Discussion

**Figure 1:**
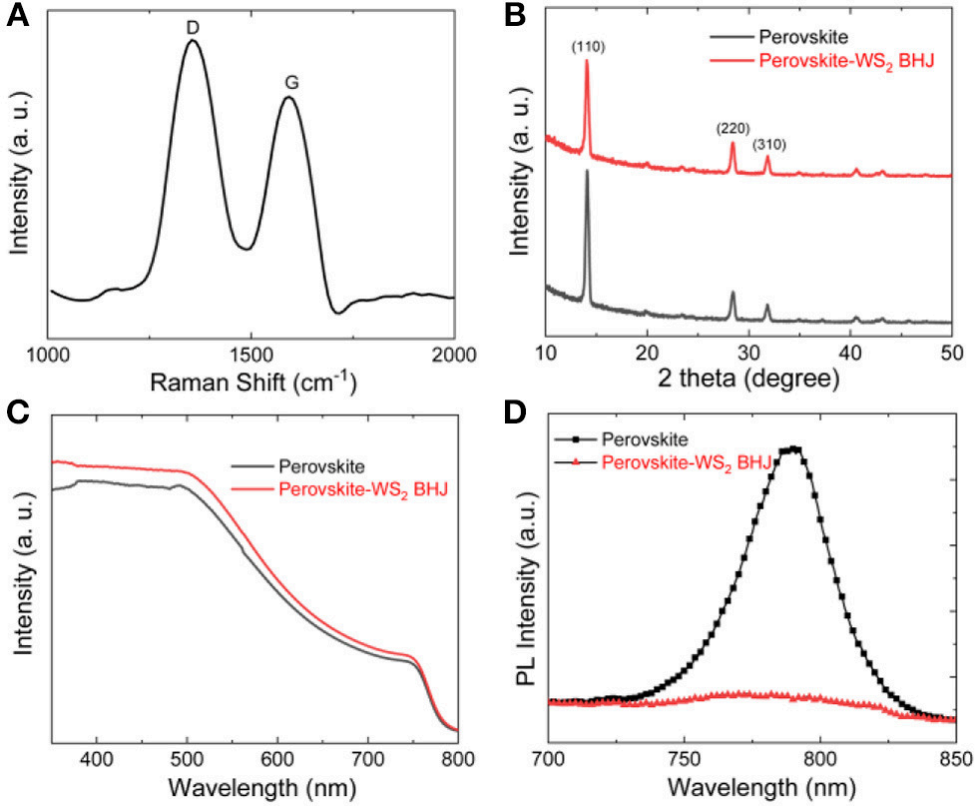
**(A)** Raman spectrum of the rGO layer, The **(B)** XRD patterns, **(C)** UV-vis absorption spectra, and **(D)** PL spectra of the perovskite and perovskite-WS_2_ composite films.

GO sheets were firstly spin-coated onto the SiO_2_/Si substrate via the monolayer APTES, and then were mildly reduced at 150°C for 30 min to obtain the rGO layer with higher electrical conductivity. Figure 1A shows the Raman spectrum of the rGO films with typical D and G bands at around 1,340 and 1,580 cm^−1^, respectively. The G band could be attributed to the graphitic carbon, while the D band is related to the structural defects or partially disordered graphitic domains (Han et al., 2015; Wasalathilake et al., 2018). The *I*_*D*_/*I*_*G*_ ratio is calculated to be 1.21, larger than the GO nanosheets, which indicates the effective reduction of GO into rGO and the partial restoration of the conjugated structures during the reduction process (Han et al., 2015; Zhu et al., 2019). Then, a one-step method was adopted to deposit the perovskite and perovskite-WS_2_ BHJ film on the rGO conductive layer, followed by evaporation of Au electrodes. The WS_2_ nanosheets exhibit a typical 2H phase, confirmed by the XRD patterns (Figure S1) (Liu et al., 2018). XRD patterns of both CH_3_NH_3_PbI_3_ and CH_3_NH_3_PbI_3_-WS_2_ composite films have similar structural features (Figure 1B), showing three main peaks at 2θ = 14.05°, 28.41°, and 31.87° associated with the (110), (220), and (310) planes of the perovskite crystals, respectively. This result reflects that the addition of WS_2_ nanosheet in the composite film has negligible effect on the crystalline structure of CH_3_NH_3_PbI_3._ The UV-vis absorption spectra of the perovskite and perovskite-WS_2_ BHJ films with the WS_2_ concentration of 0.05 wt.% is presented in Figure 1C. The perovskite films with and without WS_2_ nanosheets show broad and strong light absorption in the UV-vis region with a typical absorption edge around 786 nm. Moreover, the absorption intensity and also the spectrum profile are similar for the pristine CH_3_NH_3_PbI_3_ and CH_3_NH_3_PbI_3_-WS_2_ composite films, indicating that such a low concentration WS_2_ nanosheets contribute little to the light absorption properties of perovskite films. However, although the light absorption properties are similar, the PL peak at 776 nm arising from the interband transition of perovskite exhibits different intensities for pristine CH_3_NH_3_PbI_3_ and CH_3_NH_3_PbI_3_-WS_2_ composite films, as shown in the steady-state PL spectra (Figure 1D). A significant PL intensity quenching in the CH_3_NH_3_PbI_3_-WS_2_ composite film is observed, demonstrating that a non-radiative kinetic process rather than the radiative recombination process is induced by WS_2_ in the perovskite. Moreover, the weak PL intensity of perovskite-WS_2_ composite film indicates that the WS_2_ nanosheets provide the composite film with an advantageous energy level alignment for efficient charge transfer and separation, beneficial for promoting the photo-generated hole-electron separation and thus improved photoresponse.

**Figure 2:**
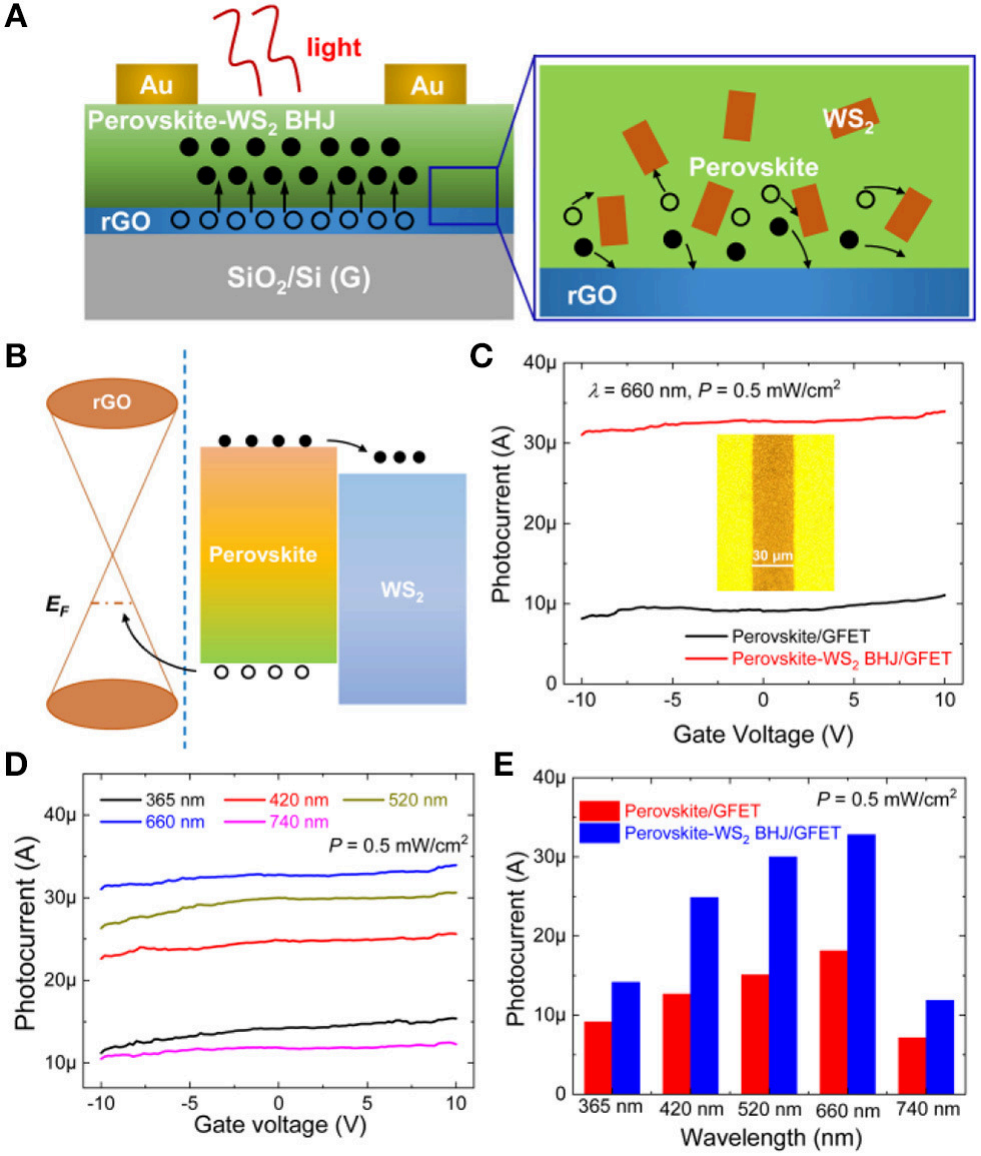
**(A)** Schematic diagram showing the photodetection mechanisms of the perovskite-WS_2_ BHJ/GFET phototransistors i.e., the electronic processes. **(B)** The energy level alignment diagram of the perovskite-WS_2_/rGO heterostructure. **(C)** The photocurrent vs. gate voltage curves of the perovskite/GFET and perovskite-WS_2_ BHJ/GFET phototransistors at a *V*_*ds*_ value of 2 V under a 660 nm illumination with a power density of 0.5 mW/cm^2^. Inset shows an optical microscope image of the device, indicating the channel length of 30 μm. **(D)** The photocurrent vs. gate voltage curves of the perovskite-WS_2_ BHJ/GFET phototransistor at a *V*_*ds*_ value of 2 V under the 0.5 mW/cm^2^ light illumination with different wavelengths. **(E)** The photocurrent value of the phototransistor with and without WS_2_ nanosheet corporation under illumination at 0.5 mW/cm^2^ with different light wavelengths (*V*_*DS*_= 2 V, *V*_*GS*_= 0 V).

Figure 2A shows the device configuration of our perovskite-WS_2_ BHJ/rGO hybrid phototransistor. Upon illumination, electron and hole pairs are generated. The significant PL quenching (Figure 1B) demonstrates that the CH_3_NH_3_PbI_3_-WS_2_ bulk heterojunction (BHJ) can effectively separate the photo-generated holes and electrons, and the electrons are transferred to WS_2_, resulting in the significant suppression of the electron-hole recombination in the perovskite. Moreover, as photo-generated electrons are trapped in the BHJ film, only holes are transferred to the rGO channel owing to the energy barrier between the perovskite and rGO film. Therefore, more negative charges will build up in the CH_3_NH_3_PbI_3_-WS_2_ BHJ photosensitizer layer, and this will induce more hole doping in rGO, which improves the photoresponse properties. The energy level alignment diagram of the perovskite-WS_2_ BHJ/rGO hybrid device structure and the corresponding electronic processes are depicted in Figure 2B according to the reported energy levels of perovskite, WS_2_ and graphene (Ma et al., 2016; Zhang et al., 2017). As a result of different Fermi levels and energy levels of CH_3_NH_3_PbI_3_ and WS_2_, photo generated electrons are transferred to WS_2_ and then holes and electrons are separated, which reduces the charge recombination within perovskite and thus enhances the photodetection performances.

To demonstrate the hole doping effects in rGO, the transfer curve of the phototransistor with and without WS_2_ nanosheets at *V*_*ds*_ = 2 V is measured in dark and upon illumination of monochromic light with different wavelengths (365, 420, 520, 660, and 740 nm) and light intensities. Figure 2C shows the photocurrent vs. gate voltage curves of the perovskite/GFET and perovskite-WS_2_ BHJ/GFET phototransistors under light illumination (λ = 660 nm, *P* = 0.5 mW/cm^2^). Inset of Figure 2C exhibits the optical microscope image of the device, and it can be clearly seen that the channel length is measured to be 30 μm. Here, the photocurrent is defined as the difference between the current under light illumination and dark conditions. The photocurrent can be modulated efficiently by altering the gate voltage, indicating the gate modulation effects on the photoresponse behaviors. Moreover, the hybrid perovskite-WS_2_ BHJ/ GFET device shows a greatly enhanced photocurrent value at *V*_*GS*_ ranging from −10 to 10 V compared with the perovskite-only device, confirming the roles of perovskite-WS_2_ BHJ on promoting the electron-hole separation and electron trapping effects. Especially, at *V*_*DS*_ = 2 V and *V*_*GS*_ = 0 V, the photocurrent of the perovskite-WS_2_ BHJ/GFET phototransistor is 32.79 μA, almost 3.5 times of that of the perovskite/GFET phototransistor (9.06 μA). This high photocurrent value is attributed to the electron-hole separation promotion effects of the CH_3_NH_3_PbI_3_-WS_2_ BHJ, high optical absorption and light-to-electron conversion efficiency of perovskite and the high carrier mobility of rGO channel layer. Figure 2D presents the wavelength dependence of the photocurrent vs. gate voltage characteristics in the perovskite-WS_2_ BHJ/GFET phototransistor at *P* = 0.5 mW/cm^2^ and *V*_*DS*_ = 2.0 V. The hybrid phototransistor exhibits different photoresponse behaviors to monochromic light illumination with different wavelengths i.e., the maximum photocurrent can be obtained under 660 nm wavelength and decreases under light illumination with longer and shorter wavelengths. The decrease of photocurrent at long wavelengths is attributed to the reduced long-wavelength light absorption of perovskite as shown in Figure 1C, while the photocurrent decrease at short wavelengths is possibly due to the low light-to-photon conversion efficiency of perovskite at short wavelengths. Moreover, it can also be seen that the gate modulation effects on photocurrent can be observed under illumination with all wavelengths (365, 420, 520, 660, and 740 nm). The photocurrents of the pristine perovskite/GFET and composite perovskite-WS_2_ BHJ/GFET devices under light illumination with different light wavelengths and the same power density (0.5 mW/cm^2^) are extracted and shown in Figure 2E. Compared with the pristine perovskite/GFET phototransistor, the photocurrents are greatly increased at all the wavelengths upon the addition of such a low concentration WS_2_ nanosheets. Especially, the photocurrents under the visible light illumination (420, 520, and 660 nm) increase more obviously than the light illumination cases with other UV and NIR wavelengths, indicating the photoresponse enhancement should be attributed to the perovskite-WS_2_ BHJ effect rather than the light absorption enhancement in the perovskite-WS_2_ BHJ/GFET phototransistor.

**Figure 3:**
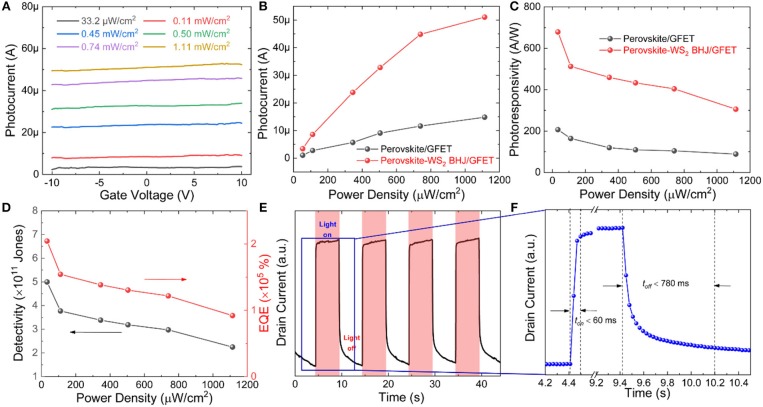
**(A)** The photocurrent vs. gate voltage curves of the perovskite-WS_2_ BHJ/GFET phototransistor at 660 nm light illumination with different power densities from 33.2 μW/cm^2^ to 1.11 mW/cm^2^. **(B)** Photocurrent, **(C)** photoresponsivity and **(D)** detectivity, and EQE values vs. incident light power density curves of the perovskite/GFET and perovskite-WS_2_ BHJ/GFET phototransistors at 660 nm light illumination (*V*_*DS*_= 2 V, *V*_*GS*_= 0 V). **(E)** Photo-switching characteristics of the perovskite-WS_2_ BHJ/GFET phototransistor measured alternately in dark and at 660 nm light illumination (0.5 mW/cm^2^) at *V*_*GS*_= 0 V and *V*_*DS*_= 2 V. **(F)** Magnified view of a single on-off cycle.

The light power dependences of the photoresponse behavior in the perovskite-WS_2_ BHJ/GFET phototransistor are further characterized. Figure 3A shows the photocurrent vs. gate voltage curves of the perovskite-WS_2_ BHJ/GFET phototransistor at 660 nm light illumination with power densities from 33.2 μW/cm^2^ to 1.11 mW/cm^2^. It can also be observed that the photocurrent could be modulated efficiently by the gate voltage, and the dependence of the photocurrent on the gate voltage increases at higher incident light power density. The extracted photocurrent value at *V*_*DS*_ = 2 V and *V*_*GS*_ = 0 V increases linearly with the increase of the incident light power density at the low power density region, and then becomes saturated in the high light power density region owing to the increased photo-excited carrier recombination rate (Figure 3B). Besides, the photoresponsivity decreases greatly with increasing the incident light power density (Figure 3C). Significantly, the photodetection performances of the perovskite-WS_2_ BHJ/GFET phototransistor including photocurrent and photoresponsivity are much superior to that of the perovskite/GFET phototransistor, demonstrating the key role of the perovskite-WS_2_ BHJ on photoresponse enhancement. At *V*_*DS*_ = 2 V and *V*_*GS*_ = 0 V, a high photoresponsivity of 678.8 A/W at an incident power density of 33.2 μW/cm^2^ is achieved in the perovskite-WS_2_ BHJ/GFET phototransistor, which is about 3 times larger than that of the pristine perovskite/GFET phototransistor (207 A/W). Even higher photoresponsivity could be achieved if the incident light with lower power density or a larger bias voltage is adopted, however, this high photoresponsivity performance is high enough for the practical applications. Moreover, it should also be noted that such high photoresponsivity is achieved at such a low operation voltage (*V*_*DS*_ = 2 V and *V*_*GS*_ = 0 V), which makes it more promising for practical applications.

To further evaluate the photodetection performances of the phototransistor, other important figures of merits such as specific detectivity and external quantum efficiency (EQE) are also calculated. The specific detectivity (D^*^) enables the device performance comparison between photodetectors with different device geometries and photosensitive areas. The shot noise is assumed to be mainly from the contribution of dark current and thus the specific detectivity can be calculated according to the following formula:

$$\begin{array}{l} D^{*}=\frac{R{\left(A\right)}^{1/2}}{{\left(2qI_{dark}\right)}^{1/2}} \end{array}$$

where *R* is the photoresponsivity, *A* is the photosensitive area, *q* is the electric charge and *I*_*dark*_ is the dark current. The calculated detectivity vs. power density curve is depicted in Figure 3D, and the detectivity shows similar dependence on power density as photoresponsivity. The maximum D^*^ value at an incident power density of 33.2 μW/cm^2^ is calculated to be 4.99 × 10^11^ Jones, which is comparable with commercial Si photodiodes (~4 × 10^12^ Jones) (Gong et al., 2009). Besides, EQE is also calculated using the following formula:

$$\begin{array}{l} EQE=\frac{Rhc}{\lambda q} \end{array}$$

where *hc*/λ represents the photon energy, *R* is the photoresponsivity and *q* is the electric charge. The calculated EQE vs. light power density value is presented in Figure 3D, and it is clearly seen that the EQE value also increases with decreasing the light power density. Notably, the perovskite-WS_2_/GFET phototransistor also exhibits much higher *EQE* values than the pristine perovskite/GFET phototransistor, and the highest EQE reaches a remarkable value as high as 2.04 × 10^5^%.

Except for the above photoresponse parameters, the photoresponse speed is also an important parameter for photodetectors. Figure 3E shows the temporal photocurrent response of the perovskite-WS_2_ BHJ/GFET phototransistor upon illumination with a 0.5 mW/cm^2^ 660 nm light at *V*_*DS*_ = 2 V and *V*_*GS*_ = 0 V. The light on/off time interval is set as 5 s. The phototransistor exhibits highly stable and reproducible photoresponse behaviors. The rise or fall time was measured to be <60 or 780 ms (Figure 3F), revealing its relatively fast photoresponse rate. However, a slower decay process is observed when the light is turned off, which is possibly due to the trapping effects of the interface states or film defects on the photo-generated carriers.

## Conclusions

In conclusions, the perovskite-WS_2_ nanosheet composite optical absorber was deposited on reduced graphene oxide to construct high-responsivity perovskite-WS_2_/rGO phototransistors via a facile and cheap solution-processing strategy. The WS_2_ nanosheets are well-dispersed within the perovskite matrix by a mixed perovskite-WS_2_ precursor, forming the perovskite-WS2 bulk heterojunction. The hybrid phototransistor shows outstanding photoresponse behaviors including high responsivity of 678.8 A/W, high specific detectivity of 4.99 × 10^11^ Jones and ultrahigh EQE value of 2.04 × 10^5^%, which is about 3.5 times of those of the perovskite/GFET phototransistor without WS_2_ nanosheets. Moreover, it also exhibits excellent photoresponse-irradiance dependence characteristics and rapid response to photoswitching. The excellent photodetection behaviors of the hybrid phototransistor is attributed to the WS_2_ nanosheets induced photo-generated electron-hole separation promotion effects due to the selective electron trapping effects in the WS_2_ nanosheets, together with the high carrier mobility of the rGO channel. The solution-processing fabrication method along with its outstanding photoresponse behaviors makes the perovskite-WS_2_ BHJ/GFET phototransistor promising for future practical photodetection applications.

## Author Contributions

R-MY designed the experiments. D-DZ conducted the experiments, analyzed the data and wrote the manuscript.

### Conflict of Interest Statement

The authors declare that the research was conducted in the absence of any commercial or financial relationships that could be construed as a potential conflict of interest.
