# Supplementary material for: Perovskite-WS2 Nanosheet Composite Optical Absorbers on Graphene as High-Performance Phototransistors
Source: Front Chem. 2019 May 8;7:257. doi: 10.3389/fchem.2019.00257 (PMC6518953; doi:10.3389/fchem.2019.00257)
Supplement: Supplementary file 1 [file Data_Sheet_1.PDF]

Supporting Information

Perovskite-WS_2 Nanosheet Composite Optical Absorbers on Graphene as High-Performance Phototransistors

Dan-Dan Zhang[1], Rong-Mei Yu[2]*

[1]Institute of Functional Nano & Soft Materials (FUNSOM), Jiangsu Key Laboratory for Carbon-Based Functional Materials & Devices, Soochow University, Suzhou, Jiangsu 215123, P. R. China

[2]College of Physics and Electronic Engineering, Nanyang Normal University, Nanyang 473061, China

** Email: yurongmei@aliyun.com*

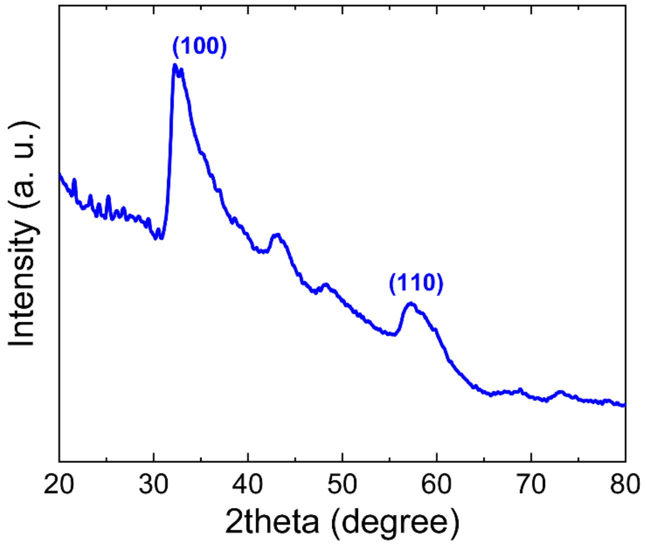

Figure S1 XRD patterns of the WS_2 nanosheets
